# Generating change through collective impact and systems science for childhood obesity prevention: The GenR8 Change case study

**DOI:** 10.1371/journal.pone.0266654

**Published:** 2022-05-11

**Authors:** Kristy A. Bolton, Penny Fraser, Janette Lowe, Marj Moodie, Colin Bell, Claudia Strugnell, Josh Hayward, Jaimie McGlashan, Lynne Millar, Jillian Whelan, Andrew Brown, Steven Allender

**Affiliations:** 1 Global Obesity Centre (GLOBE), Institute for Health Transformation, Deakin University, Geelong, Victoria, Australia; 2 Southern Grampians Glenelg Primary Care Partnership, Hamilton, Victoria, Australia; 3 Deakin Health Economics, Institute for Health Transformation, Deakin University, Geelong, Victoria, Australia; 4 School of Population Health, Curtin University, Bentley, Western Australia, Australia; 5 Institute for Health Research, University of Notre Dame, Fremantle, Western Australia, Australia; University of Toronto, CANADA

## Abstract

**Background:**

Community-based interventions have shown promise in reducing childhood overweight and obesity. However, they have been critiqued for using linear logic models. Participatory community-based systems approaches are posited as addressing the complexity of non-linear relationships in a local context. Community members are empowered to understand and describe obesity causation, identify and prioritise possible solutions. The application of such approaches to childhood obesity is in its infancy.

**Aim:**

To describe the first 12 months of a participatory whole-of-community systems approach to creating collective action to tackle childhood obesity, called GenR8 Change, in a local government area of Victoria, Australia.

**Methods:**

Three group model building (GMB) sessions focused on the development of a causal loop diagram (CLD), prioritised evidence-informed actions, and developed implementation strategies. The collective impact framework underpinned the approach, with a local backbone group supporting community members to implement prioritised actions.

**Results:**

The first two GMB sessions included 20 key community leaders where a CLD examining the factors contributing to childhood obesity in the community was constructed and refined (22 variables GMB1, 53 variables GMB2). In the third session, 171 members of the wider community further refined the CLD, identified priorities for childhood obesity prevention (72 variables in final CLD). One-hundred and thirteen individuals signed up across 13 working groups to plan and implement 53 prioritised actions. Agreed community actions included creating sugar free zones; developing healthy policies; increasing breastfeeding rates; improving drinking water access; and increasing physical activity options. Twelve months post-GMB3, 115 actions had been implemented.

**Conclusion:**

GenR8 Change is one of the first communities to apply systems thinking to childhood obesity prevention. Knowledge on how to collectively identify relevant leverage points to tackle childhood obesity can now be shared with other communities.

## Introduction

Worldwide more than 1.9 billion adults are overweight with 650 million of these persons are classified as obese [1]. Even more concerning is the extraordinarily high number (340 million) of young children overweight or obese globally [1]. In Australia, 67% of adults and 25% of children aged 5–17 years old were categorised as overweight or obese in 2017–18 [2]. The development of overweight and obesity is multifactorial, with complex drivers and individual heterogeneity in causes and consequences [3]. Childhood obesity tracks strongly into adulthood [4, 5] and increases the risk of negative long term health [6–9] and psychosocial consequences [10–12] and as such, demands a prevention approach across the life-course that has the ability to address drivers at many levels.

Community-based interventions (CBIs) have shown promise in addressing the complex problem of childhood overweight and obesity particularly by targeting key modifiable drivers such as diet and physical activity [13–15]. A recent meta-analysis and economic modelling has also revealed CBIs to be cost effective in reducing obesity in school-aged children aged 5–18 years old [16]. Community capacity and community engagement underpin the development and implementation of most CBIs [17]. Systems thinking can be used to enhance the effectiveness of CBIs by creating a shared understanding of the causes and drivers; and ways to address complex problems. Methods to examine and intervene in complex problems like childhood obesity come from a range of intellectual disciplines [18]. One approach, community-based system dynamics, appears particularly amenable to CBIs [19]. Building on and extending socioecological models, community-based system dynamics highlights the interconnections and feedback loops among actors, factors, sectors and levels of a complex issue [20] with the aim of identifying strategies for intervention sustainability, scalability and reach [3]. Community-based system dynamics seeks to identify connections within community systems and introduce new, and strengthen existing, interventions adapted to the local context that explicitly cater for non-linear relationships and unintended consequences, delays and feedback [19]. Within community-based system dynamics, community members design interventions using a collective framework and embed actions into existing systems. Consequently, the interventions are relevant to the continually changing real world [21]. While the implementation and evaluation of systems approaches to childhood obesity are in their infancy, a growing number of applications are emerging [19, 22–27].

The Whole of Systems Trial of Prevention Strategies for Childhood Obesity (WHO STOPS) [28, 29], draws on community-based systems dynamics and uses group model building (GMB) to engage and organise ten communities to prevent childhood obesity within a cluster randomised stepped wedge design. All of the communities have also used a collective impact framework [30] to ensure that solutions stem from a common shared agenda and cross-sector collaboration and co-ordination rather than individual organisations working in isolation [30].

Collective impact is defined as ‘the commitment of a group of important actors from different sectors to a common agenda for solving a specific social problem’ [30]. Five key dimensions interact which together produce a successful collective impact initiative: (i) ***common agenda*** (having a shared vision for change with a common understanding of the problem and agreeing upon joint actions to solve it); (ii) ***shared measurement systems*** (agreeing on the ways in which success will be measured and reported at the community levels across all participating organisations to ensure alignment of efforts and accountability); (iii) ***mutually reinforcing activities*** *(*conducting co-ordinated and differentiated actions from a diverse group of stakeholders—working together and mutually reinforcing the agreed plan of action); (iv) ***continuous communications*** (frequent communications (e.g. regular meetings) to build experience and motivation of all organisations’ efforts); (v) ***backbone support organisations*** (a separate organisation of skilled individuals to provide support and co-ordination for the entire initiative (e.g. facilitation of meetings, technology support, communications, data collection, reporting, administration requirements, leadership, mediation between stakeholders) [30].

The aim of this paper is to describe the process of a participatory whole-of-community systems approach to collective action for childhood obesity prevention. A case-study, GenR8 Change, from a local government area of Victoria, Australia will be presented. The key community-driven actions proposed as solutions to improve children’s eating and physical activity across the community and areas of action implemented in the first 12 months of the intervention will be presented.

## Materials and methods

### Study design and community participants

GenR8 Change is one of the WHO STOPS communities that is spread over 6,644 square kilometres in the Southern Grampians local government area (LGA) or shire in rural South-West Victoria and has a population of 16,510 residents [31]. The shire contains one large town, the main retail and service centre, and nine smaller towns. It is classified as outer regional [32] and is classified as a socioeconomically disadvantaged area compared to the national average (using census indicators such as income, education, unemployment) [33]. The main employment is agriculture, forestry and fishing (18.7%), health and social assistance (14.3%), retail trade (10.1%) and education and training (8.0%) [34]. The target population for intervention actions was primary school students from the community as previously described [28, 35]. The data on student health and wellbeing outcomes will be reported elsewhere at the end of the intervention period in 2021 [36]. In this study, the participants were the community leaders and members who designed and implemented the interventions on behalf of these children. Inclusion criteria for recruitment of participants in this study included the requirement that the participant either lived or worked in the community. The overall WHO STOPS trial has been registered in the Australian and New Zealand Clinical Trials Registry (ACTRN12616000980437).

### Summary of GMB methodology

GMB is a technique developed to support participatory systems thinking [19]. The GMB process provides facilitators and modellers a precise set of scripts to help community members develop a causal loop diagram (CLD) of the community’s mental model of the drivers of children’s diet and physical activity behaviours [37]. The modelling team adapted scripts for the GMBs from Scriptapedia [38] and each team member was trained to play a specific role during the GMB including meeting convener/closer, modeller, facilitator, note takers, wall builder, and a debriefer. Two recorders took notes to ensure that context of the variables and connections were not lost during the CLD development. A CLD is a qualitative non-linear representation of the drivers, feedback mechanisms and delays that dictate the obesogenic system [19]. Vensim [39], a free software program designed to build CLDs [22] was used throughout the live GMB workshops so that participants could see what was being created. From the visualisation of the CLD, potential leverage points to intervene in the system were identified [40] by the participants. For the full and in-depth details (roles, scripts, activities) related to conducting GMB1-3 please refer to the facilitation manual (S1 Appendix).

### Pre-intervention community context prior to GMB workshops

Concurrently, numerous complementary events within the community were occurring. Firstly, Primary Care Partnerships (PCP) and Municipal Public Health and Wellbeing planning in the shire identified that existing approaches to the prevention of childhood obesity were ineffective and a new approach was needed. Secondly, a systems approach to obesity was being trialled in Portland, SEA Change Portland (in the neighbouring Glenelg Shire) in 2014. Thirdly, the awarding of a Western Alliance Grants in Aid project enabled the establishment of a sustainable childhood obesity and risk factor surveillance/monitoring system across south-west Victoria among primary school students [28, 35]. The availability of localised risk factor and weight status data among children was key to timing the commencement of GenR8 Change and could be used to engage leadership and empower the community to action. Lastly, a new Chief Executive Officer (CEO) commenced at the Western District Health Service (WDHS) in August 2014, with an understanding of the merits of taking a new approach to obesity prevention given concerns about the ‘business as usual’ approach. The CEO also took a broader view of the local health services’ role in health prevention and in the community.

### Catalyst and initiation of GMB process

SEA Change Portland, a newly implemented community-based systems approach to address childhood obesity was demonstrating success in empowering the community to make changes to the prevent obesity [22, 41–43]. In this case, a catalyst can be defined as an individual who activated a new partnership and new information on a community health issue [44]. The catalyst in SEA Change Portland also worked at the PCP encompassing both Glenelg and Southern Grampians LGAs. The catalyst initiated discussions with the new CEO of WDHS and senior manager of the Southern Grampians Shire Council regarding SEA Change Portland and the community-based systems approach to address childhood obesity. Through existing relationships and partnerships, the catalyst identified a group of leaders whose work would align with taking this approach in the Southern Grampians LGA. GenR8 Change (whilst not officially known as this as yet) had begun. Further, to support expansion within Southern Grampians, researchers at the Global Obesity Centre, Deakin University, applied for funding from the National Health and Medical Research Council (NHMRC) through a Partnership Project Research Grant with key agencies from Southern Grampians and neighbouring areas agreeing to partner in this work.

### GenR8 Change data session with ambassadors, 12 August 2015

The catalyst put together an informal working group to begin recruiting community leaders. The community leaders (ambassadors) were identified as persons of importance, reach, influence and authority to affect the food and activity environments for children within the community. This included leaders from key service providers e.g. health service, local government, shire councillors, business owners and sporting groups. Drawing from pre-existing relationships with these community leaders, the catalyst and informal working group invited these leaders to a breakfast held at a local café where new data about the weight status and associated risk behaviours of children in the community were presented. The session was repeated again at a briefing for Southern Grampians Shire Councillors on this day.

### Group Model Building 1 (GMB1), 27 August 2015

Ambassadors and community members were subsequently invited to attend a 90 minute GMB workshop held at a local cafe where the childhood monitoring data were presented, and the case for preventing obesity in childhood explained. The question framing the workshop was “what drives children’s health in Southern Grampians?” The activities in this workshop included graphs over time (participants graphed factors that affect or are affected by childhood obesity in Southern Grampians), and connection circles (the content of the graphs over time (now called variables) were built into a connection circle as they were shared by participants. Participants then identified connections between the variables, and also added more variables as required. This work formed the first iteration of the CLD whereby a map was created using Vensim [39]. The recorded notes taken throughout the GMBs became a critical component of the map review and clean up that occurred between GMB1 and GMB2, to ensure that all detail from the workshop was documented correctly. The CLD was cleaned up and modified based upon the notes and systems dynamics conventions. The process was similar to that carried out in the pilot SEA Change Portland project [22, 41–43].

### Group Model Building 2 (GMB2), 17 September 2015

In this 90 minute workshop held at a local cafe, ambassadors revised the work from GMB1. Prior to GMB2, the map had been refined by a Deakin University systems researcher to improve readability and add variables and connections that were recorded by the note takers in GMB1. The CLD was presented back to the group and the participants were asked to confirm the modifications represented their work in GMB1; and then further refine it, by paying attention to what they felt was inaccurate or overlooked, misrepresented, required more discussion or separation into additional variables. For example, multiple variables from GMB1 addressed ‘junk food’, but participants decided the variable name ‘unhealthy food’ was a better representation of the issue. At the conclusion of GMB2, participants who had previously been confirmed as ambassadors were asked to consider who else in the community should be involved in identifying and implementing changes across the community. The ambassadors were asked to help recruit these broader members of the community to the next GMB session. Notes were taken during GMB2, which were then used post-workshop to clean up and modify the CLD as described for GMB1 above.

### Group Model Building 3 (GMB3), 14 October 2015

The third workshop (GMB3), held at a local reception centre, was open to ambassadors and the broader community. The community-led recruitment for this workshop included public invitations (radio and print media releases, Facebook, posters, etc.) and personalised invitations through email and telephone. Participant inclusion criteria was the need to either live and/or work in the community. In this half-day workshop, participants were presented with the work from GMB2 to confirm any modifications to the CLD made post-GMB2 by the modeller. Once the CLD was confirmed by the community to be an accurate representation of the key influences on childrens’ health in their community, the participants moved on to a series of action planning/prioritisation tasks. As GMB3 involved primarily a new cohort of participants, the context to the issue (i.e. childhood obesity in Southern Grampians) was presented, along with a presentation by the modeller detailing how the CLD was developed. Participants received a large-sized (A0) printed copy of the CLD and were asked to provide feedback using markers and post-it notes with the opportunity to add or remove connections and variables.

Best practice evidence regarding childhood obesity prevention and intervention strategies were presented. Referring to the CLD, participants were asked to identify as many action ideas as possible to improve childhood obesity by asking the question “how can we improve the health of children in Southern Grampians?” Working in groups, these ideas were prioritised based on feasibility and likely impact. An intervention-level framework which includes five levels of system action were used to support prioritisation of likely impact [45]. The priority actions were then organised into themes identified by the facilitator at the workshop; and confirmed by the wider group. Task teams were formed by participants signing up to the actions and theme areas they had interest, remit and capacity to commit to. The conclusion of the third GMB was considered the start of the intervention.

### Community workshops, 28 October and 4 November 2015

Two additional community workshops held at the local performing arts centre following GMB3 aimed to move the community to collective action. The previous themes were introduced and participants were invited to work within a theme, with “open space” guidelines were used, whereby people could move around as they pleased [46]. Participants developed plans for moving concept actions into reality. During this time the NHMRC announced that the Partnership Project Research Grant was successful to begin in 2016. This funding gave certainty to support Southern Grampians over the following 5-years and continuation of the childhood obesity monitoring on a biennial basis.

### Actions 12 months post-GMB3

Actions implemented by GenR8 Change in their community 12 months post-GMB3 were collected by community members at a subsequent community workshop held in a function room at the local golf club by placing a dot on the relevant variable to which the action was directed at.

### Impact data and analysis

The primary outcome of the overall WHO STOPS trial was change in childhood BMI z-scores and obesity prevalence over time [28, 36]. Secondary outcomes included changes in the following: diet quality and physical activity in primary school-aged children; school environments; social network analysis [28], and community readiness to change [47]. The outcomes of this current study are related to the process outcomes—i.e. the CLDs created, the actions and themes identified and action in GenR8 Change 12 months post-GMB3. Details for data analysis after each workshop are listed above at each relevant GMB workshop. An analysis of the GMB scripts, meeting agendas, meeting minutes and reports was undertaken to align GenR8 Change activities against the five dimensions of collective framework [30] in a narrative.

### Ethical approval

This study was approved by Deakin University Human Research Ethics Committee (HEAG-H 155_2014). Participants were briefed at the beginning of workshops regarding what their participation would involve and were given a plain language statement and consent form to make an informed and voluntary decision regarding participation. Consent was obtained in written form.

## Results

**Fig 1:**
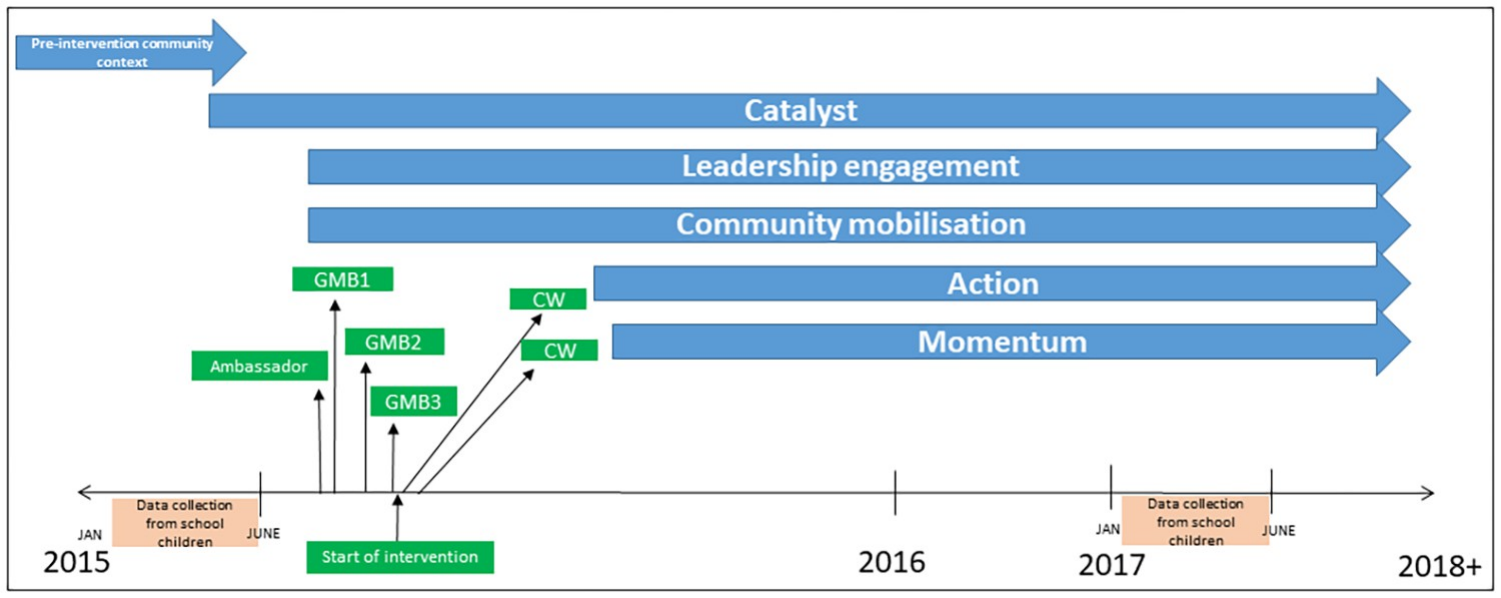
GenR8 Change process over time. Ambassador: data session for ambassadors; GMB: Group model building session; CW: community workshop.

The GenR8 Change process over time is depicted in Fig 1. GMB13 sessions were conducted over a 3-month period through August-October 2015.

### Leader engagement

#### Capacity for GenR8 Change

WDHS was aware of SEA Change Portland and saw value in aligning efforts across the region. Therefore, WDHS provided full commitment to GenR8 Change by reorienting existing resources to fund a 1.0 full-time equivalent (FTE) position to act as a conduit between community development, action and existing prevention working groups. The local council also committed to the initiative, however was not in a position to allocate dedicated staff. Backbone support [30] to enable broader collective agencies to work together was provided by the PCP (with an allocation 0.5 FTE per fortnight contributed by two staff members). The working group involved seven community members whose roles ranged from project officers through to directors, drawn from a range of settings including the health services, primary care partnership and the shire council. Members allocated two hours per fortnight for meetings.

#### GenR8 Change data session for ambassadors, 12 August 2015

There was strong community interest exhibited in the outcome results of the childhood monitoring.

Five members of the working group and 15 community leaders attended. The roles of key leaders and their organisations varied. There were executive officers (11%), directors (18%), managers (18%), councillors (11%), and medical and health professionals (11%). The remaining 31% included local business owners, chairs, project officers and staff members of organisations. The organisations the key leaders represented included the local shire council (representing 15% of the overall group), health and medical services (35%), PCP (15%), state government (5%), local and regional sporting organisations (10%), employment agency (5%), and the education sector (15%). Fourteen of these community leaders agreed to formally commit in writing to become GenR8 Change ambassadors, meaning they were committed to joining the GenR8 Change movement and had an authority to act as leaders for system change in the community.

#### Group Model Building 1 (GMB1), 27 August 2015

**Fig 2:**
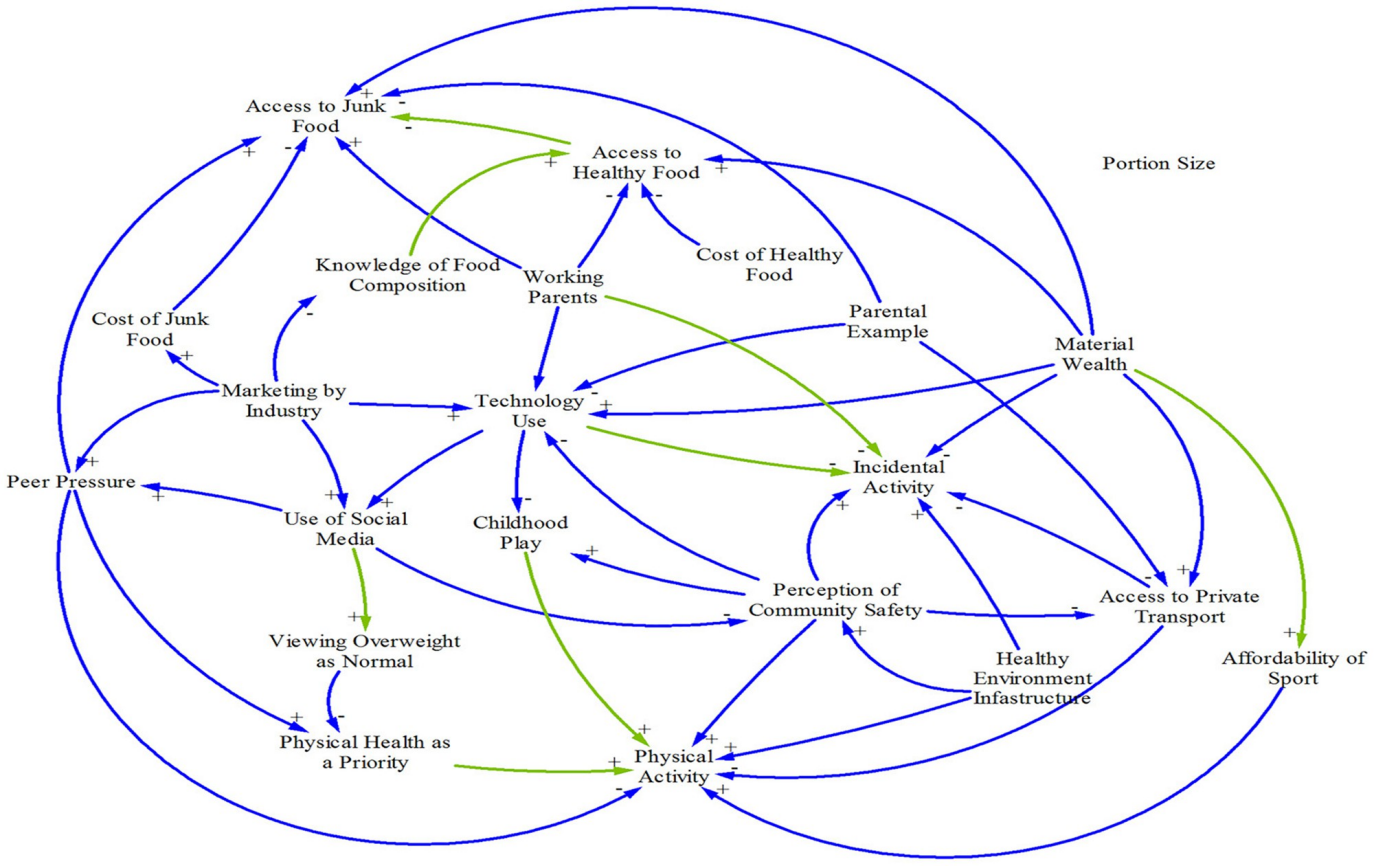
Causal loop diagram developed in GMB1. Note: blue lines are connections made in real time during the GMB session; green lines are the new connections added post-GMB session when reviewing the electronic notes taken by the recorders in the room.

GMB1 and the subsequent model review processes informed the first iteration of the CLD development (Fig 2). There were 22 variables in the CLD decided by the community leaders as key factors influencing how children eat and play.

#### Group Model Building 2 (GMB2), 17 September 2015

**Fig 3:**
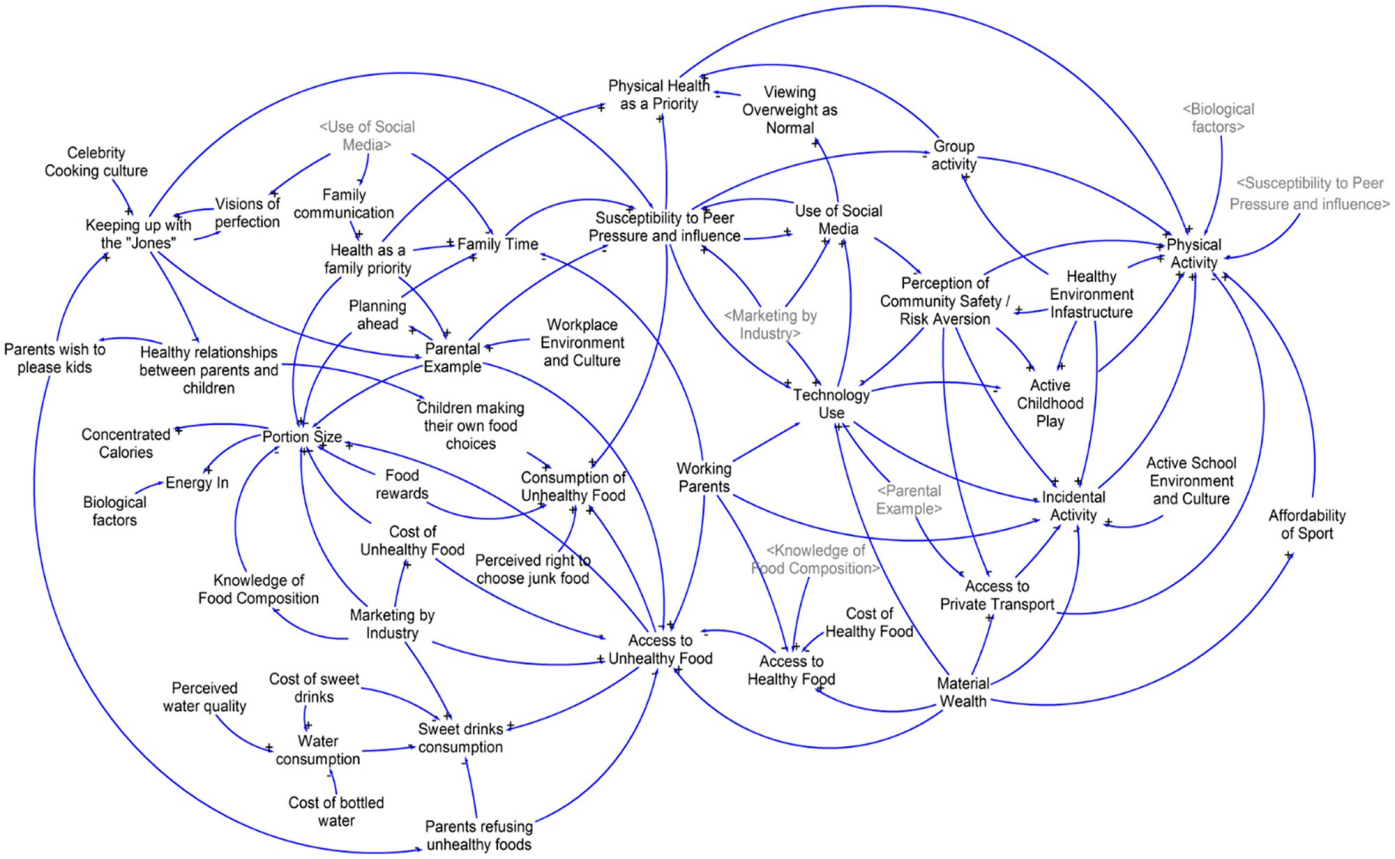
Causal loop diagram developed in GMB2.

A number of new variables were added by participants in GMB2 (Fig 3). The new variables were related to water and sugar sweetened beverage consumption, family and social norms related to diet and physical activity, unhealthy food consumption, group exercise activity and physiological drivers. New connections were also formed between variables in relation to physical activity, unhealthy food access and portion sizes, and social and media influence. In this iteration of the CLD there were 53 variables determined by the community leaders to influence how children eat and play.

### Community mobilisation

#### Group Model Building 3 (GMB3), 14 October 2015

**Fig 4:**
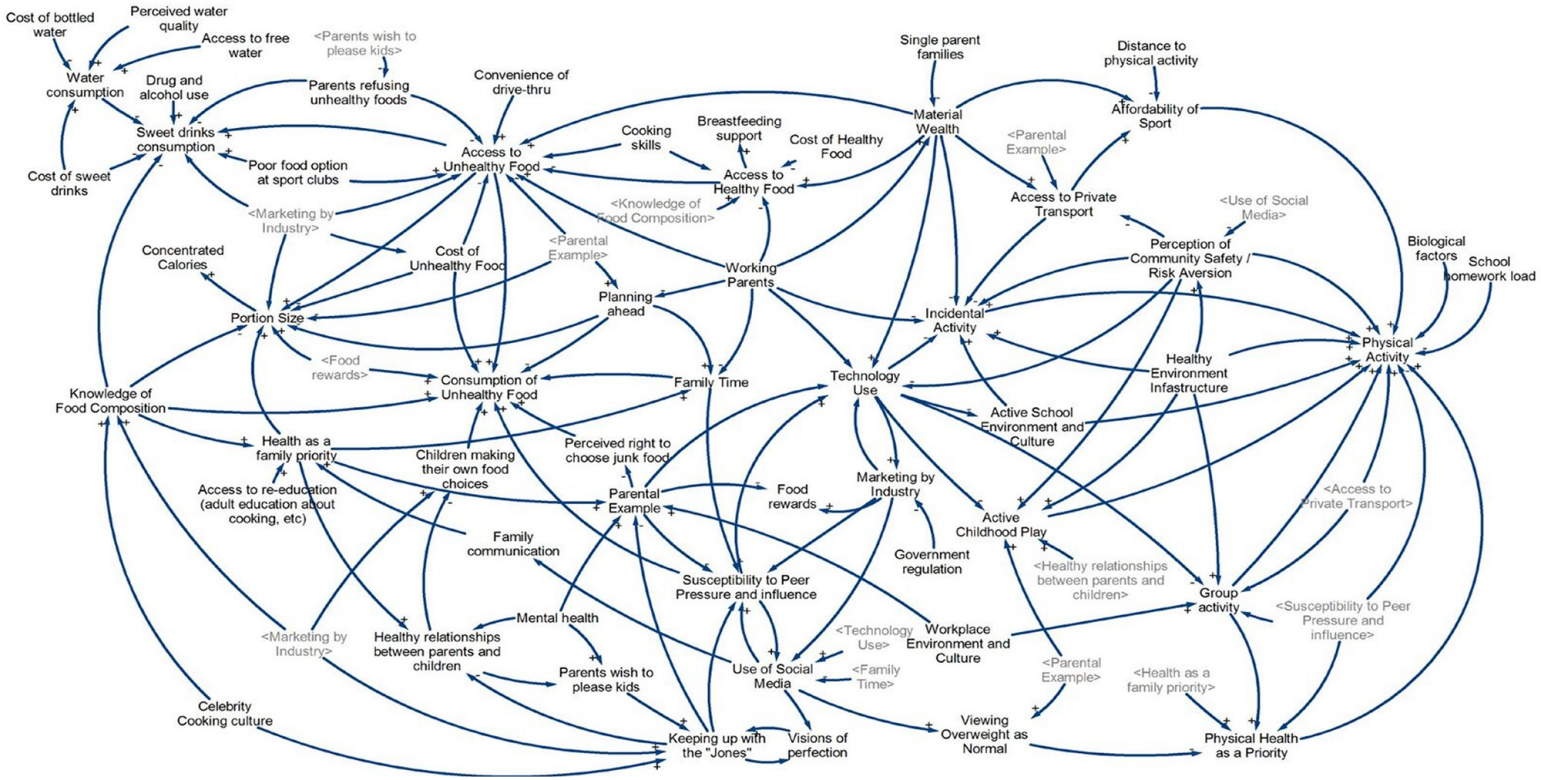
Causal loop diagram developed in GMB3.

In GMB3, 171 participants were presented with the work from GMB2 before moving on to a series of action planning/prioritisation tasks. Variables added from this workshop were: mental health, cooking skills, quality of food options available at local sports clubs and support for breastfeeding. The discussion broadly focused on the following themes: food literacy and access, exposure to technology and marketing messages, home environments and physical activity opportunities in the community. In this final iteration of the CLD, there were a total of 72 variables determined by community leaders and members as influencing how children eat and play (Fig 4).

Participants were presented with the five levels of systems action [45] to support thinking about prioritisation of actions. While a paradigm shift (the highest level) was the ultimate goal and first priority, in reality, strategies aiming at the second level, that of goals (activities that focus on or change the aim of the system), were prioritised as they could have the most impact [45]. The strategies aimed at the lowest level, changing the structural elements of the system were prioritised last as many of these elements were required to change before any meaningful systems level changes were witnessed [45]. In GMB3, 53 actions were generated and organised into 13 themes (Table 1). Examples of actions included creating sugar free zones (e.g. banning sugary drinks from junior sporting clubs); creating healthy policies (for all council run/owned facilities, early childcare settings); increasing breastfeeding friendly places (including improving breastfeeding education for health professionals, forums, community feeding spaces, Australian Breastfeeding Association feeding cafes); improving drinking water access (e.g. free water at events, improving access to drinking fountains, water friendly shops (water is available to customers for free)); and increasing physical activity options (e.g. car free zones within the community and school zones). Refer to Table 1 for development of themes and actions in the community workshops. Implemented actions were funded in a variety of ways including reorientation of already existing capacity and resources, small/local grants, and via community volunteers.

**Table 1:** Themes and actions and commitment from GenR8 Change community workshop.

Theme number	Theme	Number of actions	Number of agencies committed (number of named individuals)	Actions
1	Increase the consumption of healthy food and drinks through addressing marketing, sponsorship and fundraising	3	5 (13)	#sugarfreezones; Ban sugary drinks campaign for junior sporting clubs, clubs nominate in, develop posters SGSC ‘GenR8 Change Policy’; all council run/owned facilities, user group agreements, grants, healthy catering Fundraising the healthy way for junior sporting clubs, develop resources/guides
2	Increase breastfeeding friendly places	5	5 (8)	Investigate mental health and wellbeing impact upon feeding choices Improve breastfeeding education for health professionals, Increase formal education for health workers Place Australian Breastfeeding Association breast feeding friendly stickers in local café windows Hold consumer forum on breastfeeding and maternity services Create safe community space for breastfeeding and families, a 24/7 parenting room
3	Improving mental health and wellbeing	3	5 (5)	Develop an anti-bullying program Promote the Aspire mental health first aid program, endorsement to workplaces across shire to improve understanding of mental health and wellbeing
4	Increase the consumption of water through improving access to water	4	4 (10)	Free water at events provided by a tank/bubble tap trailer Improve access to drinking fountains Develop a ‘water friendly shop’ campaign, shops are to provide free tap water in a reusable flask, shops to have a sign/sticker on window Make carrying a cup the norm, provide free "pop up cup" key rings
5	Increase the consumption of healthy food and drinks in retail and hospitality	3	3 (4)	Survey café and hotel owners as to why they serve poor food choices to children Use healthy choices as an opportunity to attract families and children to make healthy choices Write an article about obesity and way other communities are solving the problem—gaining insight from local health professionals
6	Health education of the community	6	16 (23)	Free health education/cooking classes for parents of school aged kids, as well as pensioners, seniors, individuals with disabilities Nutritional knowledge for new parents through maternal health nurse at 4/6 month appointment, cooking demonstrations, recipe book Food depository/ co-op, have access via apps and GenR8 Change website to information, recipes and tips on label reading, sharing fresh produce in the community (excess fruit/vegetables) Run a sports equipment drive, gain donations of 2nd hand items at the library Run a health expo, food options, apply for grant for this (funding for insurance sourced) Create an online directory of health professionals, SGSC website access/maintenance, GenR8 Change website/Facebook
7	Increase physical activity options for all within the community	8	13 (18)	Make Gray St a car-free precinct and replace the road with a community garden Ban cars and busses from 200m around school gates Equipment and footwear drive, donate and redistribute to people who will use them Vouchers for accessing facilities are given as rewards for team sport, e.g. free 2 hour court hire, pool Local gyms/personal trainers run local monthly physical activity that targets whole families, family boot camp, obstacle course, The Amazing Race Increase cost of parking in the central business district Develop means of structured non-competitive sport options for all ages Pop up activity opportunities at opposing community events e.g. sports at arts and culture events
8	Health education of children within education settings	5	11 (13)	Develop a recipe book with lunchbox, snack and dinner ideas, include facts e.g.—what are the physical activity and dietary guidelines for each age group, cooking on a budget Create an ‘educational group’ of dietitians, educators etc that can oversee the healthy living message being promoted in the Southern Grampians, this group can act as a contact point for information and ideas Educate midwives to talk to women in the antenatal period Organise a Vic Market educational experience for institutions across the area e.g. kinders, schools, playgroups and mothers groups Educate all Family Day Care educators on healthy eating and have them educate children and parents in their care, make healthy eating part of the Family Day Care policy
9	Increase the consumption of healthy food and drinks in education settings	5	9 (15)	Invite parents and Parent Friend’s Association groups to join the GenR8 Change group Free Foodie afternoon/evening where dietician educates parents and staff on healthy eating habits (based on research) Menu review of canteens/lunch orders in local primary schools Newsletter inserts including nutrition and healthy eating advice, tips, recipes, vitamin and mineral information Work with Wannon Water and schools to develop ‘water only’ policy and practice within all education settings across Southern Grampians
10	Increase physical activity within education settings	3	2 (4)	Make people more aware of the scope of the obesity problem, presentations, newsletters Create stronger links between early childhood services, primary schools and secondary schools Introduce active lifestyle reps into each education setting (each kinder, school)
11	Increase physical activity through active transport to/from school	5	7 (11)	Increase availability of bikes for school children, bike swap/drive, school hiring system Educate children and road users on bike safety, teach bike education from an early age Establish active transport incentives, track days and distance, prizes Improve infrastructure (e.g. bike racks) at popular activity centres Establish safe active transport routes for school zones, signage for these routes, community "safe stops"
12	Increase the consumption of healthy food and drinks in community gatherings	2	3 (4)	Make identifying healthy food options a part of the food handling course Organise a joint meeting of all community associations and run a session on healthy food choices, identify a champion of each community group
13	Increase the consumption of healthy food and drinks in sporting clubs	1	5 (5)	#sugarfreezones, (see also under theme Increase the consumption of healthy food and drinks through addressing marketing, sponsorship and fundraising’)
TOTAL		53	88 (133)	

SGSC: Southern Grampians Shire Council

#### Community workshops

The thirteen themes were introduced and participants were invited to work within a theme, however “open space” guidelines were used, whereby people could move around as they pleased [46]. Participants worked through a series of questions, prompted by the workshop facilitator, and developed plans for moving concept actions into reality. Through this process, participants identified which areas they would like to take action in, and community task teams were formed.

### Action and momentum 12 months post-GMB3

**Fig 5:**
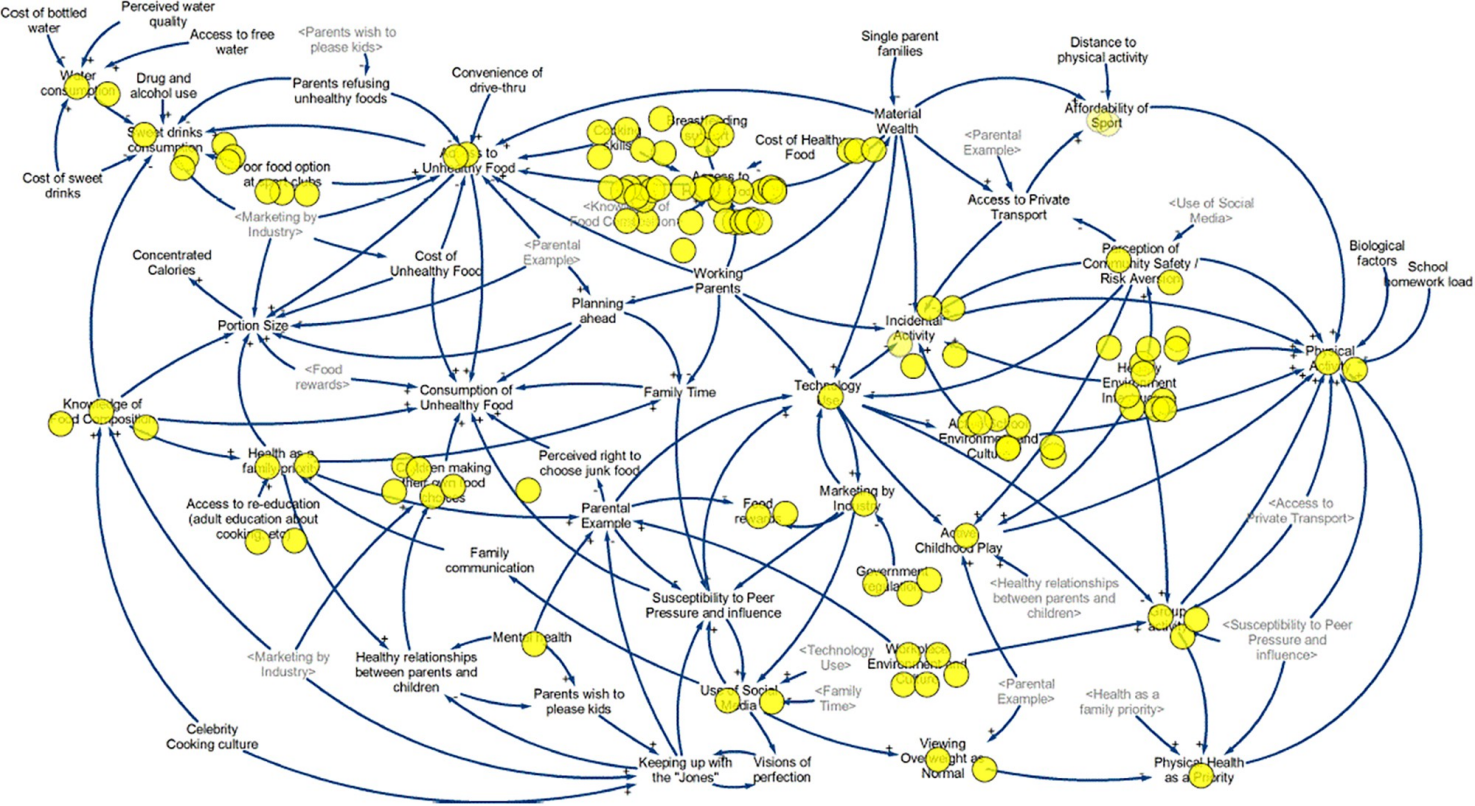
Causal loop diagram with highlighted areas of action in GenR8 Change 12 months post-GMB3.

The GenR8 Change working group continues to this day (year 2022) and is heavily involved in actions such as media, social media, meetings with task team leaders, providing advice and support to task teams, and workshops. It is not the focus of this paper to describe effectiveness of the actions presented, but rather the process of generating collective action. Actions implemented by GenR8 Change in their community 12 months post-GMB3 are highlighted on the CLD below, with each action represented by a yellow dot placed near the relevant variables (Fig 5). There were 115 individual actions in total.

### GenR8 Change underpinned by collective impact

Throughout the process multiple stakeholders from a diverse range of organisations came together with a common agenda—i.e. improving the healthiness of the community to reduce childhood obesity. Key engagement methodologies and tools throughout the process included GMB workshops; formation of working groups and implementation of actions; utilising SEA Change Portland resources; presentations by Deakin University on systems problems and solutions; Deakin University and community-led childhood obesity monitoring; and communications aligning actions to system objectives. A broad description of key activities undertaken in GenR8 Change in each dimension of the collective impact framework is now provided.

#### Common agenda

Throughout the process, a shared understanding of the problem and vision for change was agreed on [30], as demonstrated by the following activities: planning for Municipal Public Health and Wellbeing; catalyst collaborating with new health service CEO; establishment of the working group; appointment of FTE to work between the working group and community development and action; presentation of local data regarding childhood obesity prevalence and health behaviours; development of action plans; commitment of task team members; implementation of actions in the community.

#### Shared measurement

Throughout the process, data on the problem (child obesity, associated risk behaviours) and solutions [30] (i.e. actions) were collected. In addition to data related to primary and secondary outcomes, community data were collected to evaluate community implemented actions; social network analysis was conducted annually to examine change in social structures of key community leaders and their collaboration over time and economic data for cost effectiveness was collected.

#### Mutually reinforcing activities

Throughout the GenR8 Change process, community activities were differentiated yet coordinated through a mutually reinforcing plan of action [30]. Underpinning the work of GenR8 Change was planning for the Municipal Public Health and Wellbeing Plan; strategic conversations with key stakeholders; committing, implementing and evaluating a diverse range of community actions resultant from GMBs; sharing insights from SEA Change Portland; sharing success stories; and forming linkages and connections between people undertaking similar actions.

#### Continuous communication

To build trust, assure mutual objectives, and create common motivation amongst the many community players, consistent and open communication [30] in GenR8 Change occurred. The project was named GenR8 Change in order to have a brand/entity to promote community unity during communication. Other communication activities involved meetings (e.g. school principals about data collection and monitoring, with Deakin University, working group, backbone group); partnership update newsletters, the development of a common language; communication with the shire of Glenelg (SEAChange Portland); invitations to community members about workshops; social media (GenR8 Change website [48], Facebook page [49], twitter (@GenR8Change) and Instagram (#genr8change)); media and email communication.

#### Backbone organisation

A backbone for the entire initiative to coordinate participating organisations and agencies was required to achieve collective impact [30]. In GenR8 Change, locally the working group and the PCP formed the backbone. At a regional level, there was backbone support from the Great South Coast Change support group (Department of Health, Primary Care Partnership, Deakin University).

## Discussion

### Main findings

The aim of this paper was to describe the process of implementing collective action for childhood obesity prevention in GenR8 Change and assess actions 12 months post-GMB3. This study has shown how techniques from system science were applied at different stages of engaging and mobilising the community towards multiple evidence-informed strategies across the whole community with an aim of preventing and reducing childhood obesity. This coordinated community response, underpinned by the collective impact framework [30], resulted in the direct engagement of 171 individuals from the community (which included not just community members, but also 88 organisations). Consequently, 53 actions were planned for implementation in the community and 12 months post-GMB3; 115 actions had been implemented. The high attendance at GMB sessions and engagement from a diverse range of community agencies resulting in the large number of actions demonstrated that large-scale, multi-sectoral change is possible when a collective impact framework underpins a systems approach and promotes the commitment of many service providers and agencies to work with a common agenda [50]. The alignment of this work with existing core business of community leaders and members rather than adding to their workloads resulted in more “buy in” (i.e. support and participation) from the community.

#### Systems thinking and collective impact can produce community action

The union of systems thinking and collective impact is demonstrated in various ways in GenR8 Change. Pre-intervention data on weight status and associated risk behaviours from school children in the GenR8 Change community were vital in triggering the urgent need for action on childhood obesity. The data engaged and motivated community leaders to create a common agenda through the GMB process, formed the shared measurement and allowed tracking of outcomes against the actions over time to ensure the common agenda was being evaluated and reported [30]. The catalyst was an influential champion who was located in a PCP and was well connected to partner organisations such as hospitals, community health services, primary health networks, local governments, and mental health services and was also involved in the pilot project SEA Change Portland [51].

Local community ownership of the approach was crucial. A balance between community expertise and best available evidence (i.e. Deakin University support with evidence from the scientific literature) has been acknowledged as best practice in previous obesity-related community-based participatory research [52]. Furthermore, the group process (GMB sessions) which involved community-wide team development and equity in decision making and agenda setting is also considered best practice [52].

Given that obesity is a complex and multifaceted issue with heterogeneity in causes and consequences between individuals [3], mutually reinforcing and coordinated activities from a diverse range of stakeholders (community leaders and members) are needed [30]. The 53 actions in GenR8 Change were implemented across a diverse range of community settings—including retail, hospitality, sports clubs, council and education settings. Additionally, the actions were multi-dimensional and focused on improving many drivers of healthy weight such as increasing healthy food options, water access, breastfeeding, active transport and mental health and wellbeing. These actions recognised the interconnected nature of underlying systems that affect energy balance in children [3]. Economos et al. (2007) describe the need for multilevel strategies for action in the Shape Up Somerville (SUS) approach which intervened in multiple environments (e.g. school systems, before- and afterschool programs, city governments, community organizations, home environments) to provide health dietary and physical activity options; and after eight months, reduced BMI z-scores in early elementary intervention compared to comparison group school children was observed [53]. This effect was sustained with 20 month intervention data also revealing a reduced BMI z-scores in intervention compared to comparison children [54]. Similar to GenR8 Change, SUS achieved sustainability of its approach by building the actions into existing community systems from the outset; community ownership in developing and implementing actions to align with the community’s wants, needs and strengths was found to strengthen SUS actions [54].

Continuous communication and backbone support drives momentum. Similar to the Healthy Kids Healthy Communities initiative, GenR8 Change utilised a community dashboard and regularly communicated via website, Facebook page, twitter and Instagram, disseminating project communications, community partnerships, social networking and progress updates [55]. The GenR8 Change working group acted as a backbone for the entire initiative; this is required to coordinate collective impact involving planning, managing, facilitating communications, supporting and reporting to participating stakeholders and organisations [30].

### Strengths and limitations

There are many strengths in this approach. A key strength was the participatory GMB process which allowed every participant to contribute and promoted greater ownership of the actions [56]. The engagement of 171 individuals from the community in GMB3 (including 88 organisations) suggests representation of the larger community in the construction of the CLD and the design of actions to improve the health of the children locally. Systems methods allowed community stakeholders to appreciate the complexity of childhood obesity, and enabled them to design and implement multiple actions across multiple parts of the systems in their community [22]. Dividing facilitation activities into roles helped the team members efficiently manage the cognitive and practical workload involved in the GMB process [19, 22]. The systems approach was further strengthened by using a collective impact framework [30]. The number of actions implemented in the community demonstrates that multi-sector collaboration and leadership with a common agenda and mutually reinforcing activities is possible to bring about system change [50]. Sustainability is a strength of the approach, with use being made of existing community systems, capacity and resources. Sustainability of intervention action has been demonstrated; following the reduction of academic support from Deakin University after GMB3, actions are still being implemented today, years after project commencement and have expanded to over 400 actions [57]. From the point of view of capturing action and reporting on the process of implementing action, this paper is informed by practitioners both as authors and co-creators and has been written in collaboration with those working on the ground. Process data have been expressly collected prospectively to inform this descriptive piece and the causal loop diagrams have provided a type of grounded logic model to further inform these data.

Several limitations are acknowledged. Measureable outcomes (e.g. effect on health behaviours and weight status [36]) and reach of community actions have not been reported here. Not all 53 actions developed were implemented fully; some have been modified, and since 2015 more have been developed and are not presented in the current paper. Also, it would have also been beneficial to capture longitudinal effects on community stakeholder capacity building and changes in community collaboration networks using social network analysis over time. Alternate approaches to presenting implementation process stress the importance of different aspects of design and co-creation: for example, collaborative governance [58] which includes three key interactive and iterative components for collaborative action—principled engagement, shared motivation and capacity for joint action. This paper has described a process using GMB and collective impact, because the collective impact framework was identified and found to be useful by the communities themselves. By staying true to the co-creation principle with the community, we may have reduced the ability to compare this process with that of other studies. Additionally, whilst having a large attendance to GMB3 from the wider community, we cannot generalise that the CLD and actions represent all community members’ perspectives [56].

### Implications for practice

There have been numerous calls for a shift in thinking from simple, linear, causal models to examining the complex processes and outcomes that drive change within a system [59]. However, there is a scarcity of information describing the process involved in implementing a systems approach in public health, and more specifically, addressing childhood obesity. The outcomes of childhood obesity programs are generally reported, however little information exists on how these programs were accomplished [52]. Knowledge from GenR8 Change can inform the design and delivery of future systems-based approaches in community settings. The shared problem solving approach described here has revealed the importance of engaging key leaders to draw upon existing relationships to contribute to the effort; and creating and maintaining strong relationships between key community stakeholders and members [60]. Future research should focus on evaluating the sustainability of the task teams and actions implemented in the community, and whether this approach can be adapted and implemented in contextually different communities (e.g. urban localities).

## Conclusion

Through the GMB process, community leaders and members collectively identified relevant leverage points in their community system to tackle childhood obesity with successful implementation of 115 actions over 12 months. Other communities can learn from the practical insight into childhood obesity prevention action.

## Supporting information

S1 Appendix GenR8 Change GMB—Facilitation manual. (PDF)

S2 Appendix STROBE checklist cross-sectional GenR8 Change. (PDF)
