# Supplementary material for: Generating change through collective impact and systems science for childhood obesity prevention: The GenR8 Change case study
Source: PLoS One. 2022 May 11;17(5):e0266654. doi: 10.1371/journal.pone.0266654 (PMC9094504; doi:10.1371/journal.pone.0266654)
Supplement: S1 Appendix — (PDF) [file pone.0266654.s001.pdf]

GenR8 Change GMB:

Understanding Childhood Obesity in Southern Grampians, Victoria

Group Model Building Facilitation Manual V4

August-October 2015

Acknowledgements: This manual was developed by the core modeling team of Steven Allender, Lynne Millar, Melanie Nichols, Josh Hayward, Jaimie McGlashan and Brynle Owen from the WHO CC at Deakin University based on the guidance of Jill Kuhlberg and Peter Hovmand from the Social System Design Lab at Washington University in St. Louis.

Scripts presented in this manual were documented and developed through the support of numerous research grants and collaborations including the Centers for Disease Control and Prevention through the Brown School Violence and Injury Prevention Center (1R49CE001510); The Robert Wood Johnson Foundation (67099) Healthy Kids Healthy Communities evaluation project; and the Rise Sister Rise project (Ohio Department of Mental Health).

The contribution of Janette Lowe and Rosie Rowe, via GenR8 Change, is also acknowledged

GenR8 Change GMB:

Understanding Childhood Obesity in Southern Grampians, Victoria

Group Model Building Project

Table of Contents

Roles of Facilitation Team 4
Table of Facilitation Team Members and Roles 5
Detailed Agenda: Session 1 – 27/08/2015 (7:30am – 9:00am) 6
Detailed Agenda: Session 2 – 17/09/2015 (7:30am – 9:00am) 9
Detailed Agenda: Session 3 – 14/10/2015 (9:00am-2:30pm, 4:30pm-10:00pm) 11
Precise Agenda: Session 1 - 27/08/2015 (7:30am – 9:00am) 14
Precise Agenda: Session 2 – 17/09/2015 (7:30am – 9:00am) 15
Precise Agenda: Session 3 – 14/10/2015 (9:00am-2:30pm, 4:30pm-10:00pm) 16
Scripts 17
  Introduction to GMB session 18
  Graphs over time (Prioritized version) 19
  Connection Circles 21
  Community Feedback on GMB Model (Rapid Session) 23
  Live Model Update 25
  Action Ideas 27
  Prioritisation of Action Ideas 29
  Dots 31

Roles of Facilitation Team

Group model building involves successfully managing multiple roles from starting a session, to facilitating an exercise, and documenting the process. While a session could potentially be completed by as few as two experienced facilitators, the results may be compromised as the facilitators have to balance group process with the need to produce outputs using a series of structured exercises. Consequently, group model building is typically done in teams with one or more roles assigned to each team member. Below are some of the team roles needed for this project along with a description of their primary function and qualifications:

Meeting opener/closer: The meeting opener/closer convenes the meeting and brings the meeting to a close. This person is familiar with the project and its importance, and usually a recognized leader within the group. They provide a context for the overall issue and process. They do not have to be someone who was on the core modeling team or participated in the design of the sessions. The primary function of the meeting opener/closer is to start and end the meeting and set the overall stage for the group model building activities.

Modeler: The modeler is someone who is experienced in system dynamics modeling and modeling software (e.g., Vensim, iThink/STELLA) and has some experience in group model building. The modeler develops the model and helps the group reflect on model structures that emerge during the session. The Modeler also creates a tangible deliverable that participants can use during and after the session. It is important that the Modeler is familiar with the printing and production facilities, software packages being used (e.g., Vensim, iThink/STELLA, PowerPoint), and has some experience preparing printed materials involving system dynamics diagrams.

Facilitator: The facilitator is someone who has some experience in system dynamics and group model building facilitation. The facilitator works focuses on developing the diagrams, introducing concepts from system dynamics, and translating participants' statements into phrases that are easier for the modeler to use.

Note Taker: The recorder will take notes during the large group discussions. The primary function of the recorder is to document the discussion and products, and then distribute the documentation to members of the facilitation team. Sessions may have an additional recorder if needed.

Debriefer: The debriefer takes primary responsibility for convening the facilitation team after the session has been completed and lead the facilitation team through a structured debrief eliciting initial reactions and then identifying what worked and what could have been improved for the next session.

Table of Facilitation Team Members and Roles

Roles	GMB Session 1
**Convener/Closer**	Janette Lowe
**Co-Facilitators**	Steve Allender, Lynne Millar, Melanie Nichols, Jaimie McGlashan, Clinton Thomas
**Modeler**	Jaimie McGlashan, Penny Fraser
**Note Takers**	Facilitators as available
**Debriefer**	Steve Allender

Roles	GMB Session 2
**Convener/Closer**	Janette Lowe
**Co-Facilitators**	Steve Allender, Lynne Millar, Clinton Thomas
**Modeler**	Penny Fraser
**Note Takers**	Facilitators as available
**Debriefer**	Steve Allender

Roles	GMB Session 3
**Convener/Closer**	Janette Lowe
**Co-Facilitators**	Steve Allender. Group facilitators as available.
**Modeler**	n/a
**Note Takers**	Facilitators as available
**Debriefer**	Steve Allender

Detailed Agenda: Session 1 – 27/08/2015 (7:30am – 9:00am)

GenR8 Change GMB: Understanding Childhood Obesity in Southern Grampians, Victoria

Group Model Building Project

Purpose of the Session:

Participants will receive an overview of childhood obesity in Southern Grampians, Victoria. The session will transition into a group model building session that brings together stakeholders from different sectors and perspectives. Participants will collaborate, and integrate their knowledge and experience in order to identify the factors and relationships that are relevant to childhood obesity in participants' communities and practice settings.

Time	Task Duration	Activity	Description
7:00am	30 mins	*Room Setup*	Members of the GMB team arrange the room.
7:30am	10 mins	*Welcome and Introduction to GMB Session*	***The convener (Janette)*** welcomes participants and opens the meeting and provides a brief introduction to the project including available data and scope. ***The facilitator (Steve)*** gives an overview of the session and introduces facilitation team.
7:40am	35 mins	*Graphs over time script*	The ***facilitator (Steve)*** introduces the "Graphs over Time" exercise and gives participants 10 minutes to work in groups of three to draw as many graphs over time as they can on **"Things that affect or are affected by childhood obesity in your community and practice."**<br><br>At the 9-minute time limit approaches the *facilitator* gives a 1 minute warning and tells participants to prioritize graphs over time from most favorite (on the top) to least favorite (on the bottom). Then in a round-robin fashion, the *facilitator* asks participants to share one graph over time.<br><br>The ***facilitator*** takes each graph and brings it to the ***wall-builder (Lynne)***. The ***wall-builder*** organizes the graphs over time into clusters of variables on wall. At the end of one rounds of sharing, the ***wall-builder*** summarizes emergent clusters or themes.<br><br>The ***Modeler (Jaimie)*** for the following connection circle activity pre-fills the connection circle screen with participants' graphs over time, as they are shared by participants.
8:15am	5 mins	*Dots*	The ***facilitator (Steve)*** introduces the "Dots" exercise and explains to participants the purpose of prioritizing the variables which have just been

			identified.<br><br>Participants are instructed to "**place stickers on the variables that they feel are most influential on childhood obesity in Southern Grampians.**"
8:20am	35 mins or until adequate detail.	*Connection Circles*	*The **facilitator (Steve)** introduces the connection circle script.*<br><br>*The goal of a connection circle exercise is to find the connections between different concepts or variables that contribute to or are affected by some issue—in this case childhood obesity in your community and practice.*<br><br>*We will create connections, using arrows that are drawn, showing how one factor affects another.*<br><br>*The Facilitator (Steve) walks through an example, reflecting both the direction and polarity of the relationship between the variables. The facilitator lets participants know that they can reintroduce variables which were not prioritized from the graphs over time exercise, or introduce entirely new variables as they become relevant.*<br><br>The ***modeler (Jaimie)*** draws the links as the facilitator is speaking, and Vensim is projected.<br><br>*The facilitator (Steve) says that we are going to proceed in round-robin fashion around the group. Please pick two variables from this list or feel free to add another, then describe how the first influences the second.*<br><br>With 10 minutes to go, the ***modeler (Jaimie)*** will give the ***facilitator (Steve)*** an indication that the end of the session is approaching. The facilitator will ask participants if there are any last links they would like to add and closes the activity.
8:55am	5 mins	*Closing*	The ***facilitator (Steve)*** then reflects briefly on the session and explains the next steps including going over the notes and cleaning up the diagram to continue to build on the next meeting.<br><br>The ***closer (Janette)*** thanks participants for their time, invites them to stay after if they have more questions, reminds them of the deliverables that will be produced for the next workshop, and reminds them of the next meeting time/date.
9:00am	n/a	*Close Session*	Session finished.

9:00am	10-20 mins	*Debrief*	The ***debriefer (Steve)*** leads the facilitation team through a debriefing of the group model building exercise.

Detailed Agenda: Session 2 – 17/09/2015 (7:30am – 9:00am)

GenR8 Change GMB: Understanding Childhood Obesity in Southern Grampians, Victoria

Group Model Building Project

Purpose of the Session:

Participants will revisit their work from the previous session and will briefly revise the diagram before moving on to a series of action planning/prioritization tasks.

**Time**	**Task Duration**	**Activity**	**Description**
6:30am	30 mins	*Room Setup*	Members of the GMB team arrange the room.
7:00am	5 mins	*Welcome and introduction*	***The convener (Janette)*** welcomes participants and opens the meeting. The vision for improved childhood obesity is reiterated to the group as a restatement of the purpose of the workshop.
7:05am	10 mins	*Model presentation:*	The ***Presenter (Lynne)*** gives a brief outline of the development of the model from last workshop's outputs to the current version of the map.
7:15am	25 mins	*Community Feedback on GMB Model*	***The facilitator*** **(*Steve*)** at the front of the room explains the purpose of the exercise, inviting participants to use sticky notes to provide different types of feedback to the new version of the model.<br><br>Participants can convey positive comments (*"things I liked"*), concerns or proposed changes (*"things I think are wrong/need to be changed"*) and comments (*"this is new knowledge/this requires further investigation"*). Positive sticky notes should be identified by a tick in the top corner of the note, concerns or changes with a cross, and general comments with a dash (–).<br><br>At this stage participants are also invited to add any links to the CLD that they think are missing, or add polarity to any connections which are missing polarities from the first version of the CLD.<br><br>***The facilitator*** **(*Steve*)** gives 30 minutes to write and display comments. With 5 minutes to spare, the ***facilitator*** instructs participants to place their remaining comments.<br><br>When the time has elapsed, the ***facilitator (Steve)*** thanks the group for their participation. Diagrams are retained for later use.

7:40am	15 min	*Problem Solving*	***The facilitator (Steve)*** explains specific problems found in the model from workshop 1. Participants discuss solutions to the problems as the ***modeler (Penny)*** is updating the current version of the map on Vensim
7:55am	30 mins	*Live Model Update*	***The facilitator (Steve)*** recaps on the issues and clarifications requested from the ***modelling team***. A short exercise is conducted where the facilitator probes the participants on variables which may need clarification, or connections which might need to be revisited.<br><br>Throughout the discussion, the ***modeler (Penny)*** is updating the current version of the map on Vensim to reflect the participants' commentary.
8:25am	n/a	*Close session*	The ***closer (Janette)*** thanks participants for their time, invites them to stay after if they have more questions, and reminds them of the date and aims of the next session.
8:45am	10-20 mins	*Debrief*	The ***debriefer (Steve)*** leads the facilitation team through a debriefing of the group model building exercise.

Detailed Agenda: Session 3 – 14/10/2015 (9:00am-2:30pm, 4:30pm-10:00pm)

GenR8 Change GMB: Understanding Childhood Obesity in Southern Grampians, Victoria

Group Model Building Project

Purpose of the Session:

Participants will revisit their work from the previous session and will briefly revise the diagram before moving on to a series of action planning/prioritization tasks. Special focus will be on fostering cross-sector relationships in addressing the issue of educational attainment in the region.

Two sessions planned – 9am to 2.30pm and 4.30pm to 10pm (session time is 5.5 hrs including setup).

Day Time	Evening Time	Task Duration	Activity	Description
8:30am	4:00pm	60 mins	*Room Setup*	Members of the GMB team arrange the room.
9:30am	5.00pm	10 mins	*Welcome and Introduction*	***Welcome to country and community leader points to the relevance and importance of the work. The convener (Janette)*** welcomes participants and opens the meeting. The vision for improved obesity is reiterated to the group as a restatement of the purpose of the workshop.
9:40am	5:10pm	20 mins	*Best practice & Evidence Part A*	The ***Presenter*** (***Steve***) gives the group an understanding of why obesity is important and the magnitude of problem in Southern Grampians and how this approach is promising to tackle it
10:00am	5:30pm	5 mins	*Model presentation:*	The ***Presenter (Jaimie)*** gives a brief outline of the development of the model from last workshop's outputs to the current version of the map.
10:05am	5:35pm	5 mins	*Reading the causal diagram*	The ***facilitator*** (***Steve***) leads a brief discussion of how to read a CLD.
10:10am	5:40pm	35 mins	*Community Feedback on GMB Model*	***The facilitator*** (***Steve***) at the front of the room explains the purpose of the exercise, inviting participants to use sticky notes to provide different types of feedback to the new version of the model. Participants can convey positive comments (*"things I liked"*), concerns or proposed changes (*"things I think are wrong/need to be changed"*) and comments (*"this is new knowledge/this requires further investigation"*). Positive sticky notes should be identified on a green post-it,

				concerns or changes on a red post-it, and general comments on a blue post-it.<br><br>At this stage participants are also invited to add any links to the CLD that they think are missing, or add polarity to any connections which are missing polarities from the first version of the CLD.<br><br>***The facilitator*** (***Steve***) gives 15 minutes to write and display comments. With 5 minutes to spare, the ***facilitator*** instructs participants to place their remaining comments.<br><br>When the time has elapsed, the ***table facilitators (all)*** lead the group through a quick debriefing exercise in table groups, around likes/dislikes from the model and variables/linkages added.
10:45am	6:15pm	20 mins	*Best practice & Evidence Part B*	***The Presenter (Steve)*** gives participants a 10-15 slides summary of current best practice and evidence regarding the community based prevention of obesity.
11:05am	NA	10mins	*Morning Tea*	Break
NA	6:35pm	20 mins	*Dinner Break*	Guests tea
11:15am	6.55 pm	40 mins	*Identifying and prioritising action ideas*	The ***facilitator (Steve)*** introduces participants to the next activity. Participants will be given slips of A5 paper, and are instructed that the next 40 minutes will be spent identifying action ideas. Participants are instructed to examine the map, and look for "areas" or "parts" of the map where we could potentially introduce an intervention to improve the outcome of obesity.
12:15pm	7.35 pm	30 mins (daytime)<br><br>20 mins (evening)	*Prioritization*	Table facilitators lead the tables who work in groups. Groups are asked to prioritise actions based on feasibility and likely impact in groups of three (top 5/group) and then for the whole table (top 3/table).<br>As they are sharing the Facilitator is checking that action ideas fit with pre-prepared action theme list, and adds to theme list for those not included.<br>Then the facilitator asks each table to provide 1 - 2 examples of priority actions discussed on one pre-prepared theme area each. Then the Facilitator asks the table Facilitators there is additional priority themes that their table discussed.<br>During this process the Scribe (***Janette***) has transferred the examples and additional priority themes onto individual butchers

				paper and placed them on the wall for the next session.
12:45	7.55 pm	15 mins	*Session closing*	***The Closer (Clinton)*** presents the participants with a list of the action ideas they prioritized at the end of the last session. The participant are asked to add their names to the priority themes they are interested in on A3 sign-up sheets at each table and invited to continue the discussion at next workshop The participants are given a quick debrief of the overall workshop process, and have a chance to share any reflections on the session, before the workshop is brought to a close.
1:00pm	8:10 pm	n/a	*Close session*	Session finished.
1:00 pm				Lunch (daytime session)
1:30 pm	8.30pm	10-20 mins	*Debrief*	The ***debriefer (Steve)*** leads the facilitation team through a debriefing of the group model building exercise.

Precise Agenda: Session 1 - 27/08/2015 (7:30am – 9:00am)

GenR8 Change GMB: Understanding Childhood Obesity in Southern Grampians, Victoria

Group Model Building Project

Purpose of the Session:

Participants will receive an overview of childhood obesity in Southern Grampians, Victoria. The session will transition into a group model building session that brings together stakeholders from different sectors and perspectives. Participants will collaborate, and integrate their knowledge and experience in order to identify the factors and relationships that are relevant to childhood obesity in participants' communities and practice settings.

**Time**	**Activity**
7:30am	*Welcome and Introduction to GMB Session*
7:40am	*Graphs Over Time activity*
8:15am	*Dots*
8:20am	*Connection Circles activity*
9:00am	*Session Close*

Precise Agenda: Session 2 – 17/09/2015 (7:30am – 9:00am)

GenR8 Change GMB: Understanding Childhood Obesity in Southern Grampians, Victoria

Group Model Building Project

Purpose of the Session:

Participants will revisit their work from the previous session and will briefly revise the diagram before moving on to a series of action planning/prioritization tasks.

**Time**	**Activity**
7:00am	*Welcome and Introduction to GMB Session*
7:15am	*Model Update*
7:20am	*Participant Feedback on GMB Model*
7:50am	*Live Model Update & Problem Solving*
8:25am	*Closing*

Precise Agenda: Session 3 – 14/10/2015 (9:00am-2:30pm, 4:30pm-10:00pm)

GenR8 Change GMB: Understanding Childhood Obesity in Southern Grampians, Victoria

Group Model Building Project

Purpose of the Session:

Participants will revisit their work from the previous session and will briefly revise the diagram before moving on to a series of action planning/prioritization tasks. Special focus will be on fostering cross-sector relationships in addressing the issue of educational attainment in the region.

**Activity**
Welcome and introduction to GMB session
Model presentation
Community feedback on GMB model
Best practice brief
Identifying and prioritising action ideas
ORID debriefing exercise
Session close

Scripts

Group model building sessions typically consist of a sequence of small group activities or “scripts”. These scripts describe the essential components of an exercise along with the inputs from other exercises needed to do the script and the outputs produced from the script. There are scripts for working directly with participants (“online” scripts) as well as scripts for the facilitation team before and after a group model building session (“offline” scripts). Additional information about scripts can be found in the latest version of Scriptapedia, available on request from the Social System Design Lab at Washington University in St. Louis.

Introduction to GMB session

**Description**	This script is used to introduce people and set the stage for a GMB session.
**Context**	At the very beginning of a GMB session as participants are starting to get settled and the team wants to begin the session
**Purpose**	To introduce the GMB session, team members, participants, and stage the activities for the GMB session
**Primary Nature of group task**	Convergent<br>Facilitator
**Time**	**Preparation**: None<br>**Session**: 10-20 minutes, depending on number of participants and complexity of session being reviewed<br>**Follow-up**: None
**Materials**	1. Agenda of session for participants
**Inputs**	None
**Outputs**	None
**Roles**	• Meeting opener with status among the participants who can start the session
**People in the room**	• Modeling team<br>• Participants
**Steps**	1. The **opener** announces the start of the session.<br>• Welcome the participants and thank them for attending.<br>• If the session is taking place in unfamiliar room, inform participants of the location of restrooms, exits, etc.<br>2. The **opener** begins the introductions:<br>• Introduce yourself, and then say that there are more members of the modeling team in the room, and before we get to the participants, we want to let you know who we are.<br>• Each team member introduces themselves and describes their role.<br>• The **facilitator** then asks participants to introduce themselves, their organization, and how they are connected to this group today.<br>• The **facilitator** then describes the plan for the modeling session, when breaks will be, and asks if all participants are ready to begin.
**Evaluation criteria**	• Participants feel oriented to session activities
**Author(s)**	Unknown
**History**	Originally documented by Timothy Hower (thower@wustl.edu), Krista Rux (krux@wustl.edu) and Peter Hovmand (phovmand@wustl.edu) for the Federal Reserve Bank Project, September 21, 2011.
**Revisions**	None
**References**	None

Graphs over time (Prioritized version)

**Description**	Participants produce sketches of key variables over time, which are clustered by the modeling team
**Context**	At the beginning of a group model building workshop when the group has not develop a dynamic perspective of the problem or the variables involved
**Purpose**	To frame the problem from a dynamic perspective and elicit variables that could be used to decide on the reference mode for the project
**Primary nature of group task**	Divergent
**Time**	**Preparation**: 10 minutes<br>**Session**: 30 minutes<br>**Follow-up**: none
**Materials**	1. Stacks of A5 white paper with axis drawn on them<br>2. Large blank wall/white board<br>3. Permanent markers<br>4. Blu Tack<br>5. Laptop with Vensim
**Inputs**	None
**Outputs**	Candidate variables for the dynamic model or causal map
**Roles**	• Facilitator to work with the group with some experience with SD<br>• Wall builder to cluster graphs and talk about themes with little or no experience in SD<br>• Recorder to document the session and photograph the clustered graphs<br>• Modeler to take notes on variables described during session.
**People in the room**	• Participants<br>• All members of the core modeling team
**Steps**	1. The **facilitator** explains the purpose of the session, and begins by giving an example of how to draw a behavior over time graph, using a pre-filled PowerPoint slide.<br>2. The **facilitator** then asks participants to work individually to draw one variable over time per piece of paper. The participants should be given the option of including hoped for behavior, expected behavior, and feared behavior on the same graph.<br>3. The **facilitator** and **wall builder** walk around and help **participants** with the task if they need it. Allow 10 minutes or until the group runs out of steam to complete the task.<br>4. Reconvene as large group.<br>**The facilitator** instructs subgroups to form groups of three, and share their graphs with each other and choose the ones they think are most important, prioritizing the graphs they have drawn, with the most important ones at the top of the pile – participants have 5 additional minutes for this task<br>5. Reconvene again as large group.<br>The **facilitator** then goes to each subgroup and holds the first graph they have selected up in front of entire group. The subgroup

	spokesperson talks about the graph. Ask subgroups to share the "best stuff" first. Clarify timescale, variable names, etc.<br>6. The **facilitator** then hands the graph to the **wall builder**.<br>7. The **facilitator** repeats steps 5 and 6 with each participant or subgroup, taking one graph at a time until a satisfactory number of graphs have been shown. Finish by asking if any participant has something else that really ought to be shown.<br>8. During steps 5-6, each graph is posted on the wall. The **wall builder** tries to cluster the graphs meaningfully on the fly, based on themes and variables.<br>9. As the variables are being clustered on the wall, the modeller is entering the variable names in Vensim (on the computer only, not yet projecting) and arranging the prioritized variables into a connection circle for the next script.<br>10. Once a list of 15-20 variables has been completed, the **facilitator** asks the **wall builder** to explain the clusters of graphs on the wall.<br>11. The **facilitator** enables the **participants** to talk about the clusters and the characterization of the problem they imply.
**Evaluation criteria**	• Interesting, self-sustaining group discussion after clusters described by the wall builder<br>• Meaningful clusters are possible to see<br>• Graphs tend to converge to a clear dynamic problem<br>• Some key dynamic variables emerge from reflecting on the graphs and clusters<br>• Modeling team can begin to see key stocks and perhaps important feedback loops<br>• Members of the group appear to have better understandings of the issues of interest to other members
**Authors**	George P. Richardson and David F. Andersen
**History**	Originally documented by George Richardson, David Andersen, Peter Hovmand, Timothy Hower and Annaliese Calhoun in February 2010
**Revisions**	Tailored to the March 5, 2014 GMB demonstration session for S65-5050 course
**References**	Andersen, D. F., & Richardson, G. P. (1997). Scripts for group model building. System Dynamics Review, 13(2), 107-129.

Connection Circles

**Description**	Connection circles help groups visualize important variables and connections between them.
**Context**	Social systems have many variables and connections relating them. Seeing all the connections is challenging and we can quickly feel overwhelmed by the complexity of a system. To address this limitation, we need visual tools that can help us see and talk about the connections in a system.
**Purpose**	• To make explicit important variables and connections between variables<br>• Eliciting important variables<br>• Eliciting linkages
**Primary nature of group task**	• **Divergent:** Groups may come up with different connections between variables by generating a variety of variables and interpretations
**Time**	**Preparation**: 5 minutes (setting up Vensim projector)<br>**Session**: 40 minutes<br>**Follow up**: 60+ minutes, depending on exercise output
**Materials**	• Overhead data projector & screen<br>• Computers running Vensim, connected to projector and a network for backup<br>• Recorder's materials (could be computer based, or handwritten)
**Inputs**	• Variables from prior work (in this case, from the Graphs over time script) typed in Vensim on the side of a large circle.
**Outputs**	• Connection Circle
**Roles**	• ***Modeler*** with some experience on Vensim<br>• ***Facilitator*** with experience facilitating groups and some experience with building models in Vensim<br>• ***Recorder*** trained to take recorder notes during a meeting
**People in room**	• Participants<br>• Modeling team
**Steps**	1. The ***facilitator*** is at the front of the room. The ***modeler*** is sitting with a laptop connected to the data projector at the side of the room. The ***recorder*** is seated on the periphery of the participant group where all members are audible.<br>2. The ***facilitator*** introduces the exercise:<br>• *The goal of our exercise is to identify the variables and connections between variables that are important in the system affecting adolescent diet and physical activity in your community.*<br>• *A connection circle is a visual tool that can help us see the connections in a system.*<br>3. The ***modeler*** projects the connection circle with variables arranged in circle on the screen. The ***facilitator*** introduces the variables as those from the Graphs Over Time activity, noting that these variable meanings may be further negotiated as the session proceeds and also that participants are free to add variables that are not on the screen, but are important to understanding the system.<br>4. The ***facilitator*** opens the exercise by stating:<br>• *We are going to proceed in round-robin fashion around the group. Please pick two variables from this list or of your own choosing, then describe how the first influences the second.* The ***modeler*** creates a Vensim drawing of what the ***facilitator*** is describing, simultaneously, for the participants

	to see. The facilitator uses language of both direction and polarity.<br><br>5. The ***facilitator*** then prompts the group by asking:<br>• *What are some connections that you can see between any two variables on the screen?*<br>• *Once a participant nominates connection between two or more variables the community facilitator needs to be sure the variable definitions and nature of the causal connection is clear. Consider prompting the participant to share how they are thinking about the variables.*<br>6. The ***facilitator*** alternate eliciting linkages and variables from participants.<br>7. As participants nominate linkages, the ***modeler*** selects the variables and draws the linkage on the screen. As the number of variables chosen grows, ***the modeler*** should expand and rearrange the circle as needed, being aware of positioning the variables such that they are not always physically adjacent. Once one complete round or approximately ten connections are made, the ***facilitator*** says:<br>• *We have a good start and a number of connections, so we don't need to continue to go around in order. Feel free to continue to suggest connections about these or additional variables that you think are important.*<br>8. The ***facilitator*** provides a 5 minute warning to the group as the session approaches a close. The ***facilitator*** indicates when there is approximately one minute left to elicit any final input.
**Evaluation criteria**	• Each participant engages in discussing linkages and variables<br>• A connection circle with multiple feedback loops is created<br>• Participants recognize there is a complex system surrounding childhood obesity in Southern Grampians.<br>• Participants enthusiastic about modeling process
**Author(s)**	Unknown
**History of Script**	Utilized in Rise Sisters Rise project July 2011
**Revisions**	May 22, 2012 Revised by Alison Kraus and Peter Hovmand for Washington University TREC 4 GMB session<br>Modified in June 2012 by the TREC4 Core Modeling Team
**References**	None
**Notes**	None

Community Feedback on GMB Model (Rapid Session)

**Context**	After a causal-loop diagram or stock-flow map has been developed
**Purpose**	To give participants time to familiarize themselves with model or diagram that has been revised offline since the last workshop.<br>(Rapid Session version not intended for work which will lead to further revision of the map)
**Primary nature of group task**	Divergent
**Time**	**Preparation**: Very large representation(s) of a current version of the model/diagram (printed or drawn) taped/affixed to walls or windows<br>**Session**: 30 minutes<br>**Follow up**: 60 minutes (post session) to incorporate participant feedback into model.
**Materials**	• Very large poster-size representation of model/diagram (printed or drawn)<br>• High quality sticky notes (high-stick)<br>• Several dark felt tip pens (one for each participant)
**Inputs**	Causal loop diagram or stock and flow diagram currently being developed in GMB project
**Outputs**	Causal-loop diagram or stock-flow map with stakeholders' anonymous comments on post-its (good = ticked; concern = crossed; neutral = dashed) attached *at the relevant place* on the diagram/model; digital photographs of map/model with post-it comments
**Roles**	• **Facilitator** to introduce the representation to the large group and introduce the guidelines for the activity
**People needed in the room**	• Participants
**Steps**	1. Based on group size, decide on how many small groups and representations are required to have made before the script begins. An ideal group size is approximately 3 participants per large representation.<br><br>2. The facilitator at the front of the room explains the overall purpose of the exercise (to gain feedback from the larger community on an interim model, having already provided a brief overview of question/focusing problem, process-to-date, and the model to the large group). Previously, the participants have been given the information required to have a useful interaction with the representation (i.e., descriptions/examples of polarity, directionality, feedback loops, etc.). Any of this information is also displayed for participants throughout the activity.<br><br>3. The ***facilitator*** then sets up what the participants will be doing in the activity, inviting them to use sticky notes to provide different types of feedback on parts of the current version of the model. Comments may be positive (things they like/agree with/see as high value), negative (things that are erroneous/need adjustment/missing from the model) or general comments (new thoughts/things to investigate further/other). The same sticky notes are used for any kind of comment, but participants are to draw a tick in the top right corner for positive comments, a cross in the top right for concerns, and a dash for general comments. Participants write a brief comments explaining their like/concern/comment using felt tip pens on the sticky notes and place them on the part of the model

	the note pertains to.<br><br>4. At the same time – the **facilitator** invites participants to add any new causal linkages to the model which they identify throughout the task of reviewing the model.<br><br>5. The facilitator gives 30 minutes to the group to write and display their comments. Any available facilitators can act as "floaters" to respond to participants' questions.<br><br>6. Participants spend time with the representation, making and placing comments. With 5 minutes to spare, the **facilitator** asks the participants to write and place their remaining comments and questions.<br><br>7. ***The facilitator*** thanks participants for their participation, and runs through a quick debrief of the exercise based on some seed questions<br><br>a. What did you feel were some good aspects of the model – what did you place ticked sticky notes on?<br>b. What did you feel were some areas that needed to be changed – what did you place crossed sticky notes on?<br>c. Were there any general comments to share?<br>d. Were there any variables that you felt needed to be added or changed in the model?<br>e. Were there any new connections identified on the model?<br><br>8. ***The facilitator*** thanks the large group for their participation and hands over to the facilitator of the next session.<br><br>9. The diagrams with participants' comments are retained for use in the following model development.
**Evaluation criteria**	Feedback received from the community on the current version of the model<br>Participants feeling they have made a contribution to current and future steps towards a shared understanding of a problem
**Author(s)**	Jill Kuhlberg & Don Greer & Laura Black
**History**	Adapted for use with an interim CLD in GSC Portland Childhood Obesity GMB Community Session: July 17, 2014
**Revisions**	
**References**	
**Notes**	

Live Model Update

**Description**	Participants are involved in a round-table discussion of the current version of the model, and updates are made in real-time
**Context**	For the purpose of updating a model from a prior workshop, in the current workshop, to reflect new understandings generated during the session
**Purpose**	• To create a revised CLD
**Primary nature of group task**	• Convergent
**Time**	**Preparation:** nil<br>**Session**: 30-40 minutes
**Materials**	• Vensim file of existing model version<br>• Projector and screen
**Inputs from other scripts**	• Previous CLD file to be updated<br>• Participants comments attached to hard-copy version of the CLD from previous activity (Community feedback on model)
**Outputs from this script**	• Revised CLD to be developed further offline
**Modeling team roles**	• **Facilitator** to lead the discussion around what needs to be added/changed/removed from the CLD<br>• **Modeler** with experience in Vensim to update the model in real time<br>• **Note takers** to capture the discussion
**People in the room**	• Participants<br>• Facilitation team
**Steps**	1. ***The Facilitator*** opens the session by reflecting that participants have just spent a block of time getting to know the most recent version of the CLD, and commenting on it, providing reactions to what is good in the model, what is interesting about the model, and what needs improvement.<br><br>2. ***The Facilitator*** instructs participants that they will now have a chance to have some of those changes made in real-time, to begin the process of revising the map for the next session.<br><br>3. ***The Facilitator*** outlines the task as being a round-robin type discussion, whereby the small groups who worked together on the model feedback task will have a chance to suggest their most important change to the model, whether it be an addition of new material, removal of old material, or alteration of existing material. Although groups will take it in turns to describe their desired changes to the model, discussion of the changes is encouraged between

	groups if other participants have something to add to the discussion***. Note takers*** capture the discussion as best they can throughout the session.<br><br>4. As ***the facilitator*** is eliciting new information from groups, and guiding the discussion in the room, ***the modeler*** captures the changes in Vensim, which is being projected on the screen in real time.<br><br>5. (OPTIONAL) – if the ***modelling team*** have any "dead buffalos" or variables which require specific attention, 5-10 minutes may be reserved to specifically query the participants on these points, if they do not naturally arise throughout the course of the activity.<br><br>6. With 5 minutes to go, ***the facilitator*** alerts the room that we are almost out of time, and that we can take two or three quick last-minute changes before the model is taken away to be revised for the next workshop,
**Evaluation criteria**	• Participants see their input incorporated into the model<br>• Participants retain ownership of an evolving model<br>• New data is obtained which can be used to further progress the model
**Author(s)**	Written by Josh Hayward and Steven Allender (Deakin University, WHOCC for Obesity Prevention)
**History**	Designed for the GenR8 Change and Vic Pol workshops (May-June 2015)
**Revisions**	Nil
**References**	Nil.

Action Ideas

**Context**	After a model has been developed.
**Purpose**	To identify potential actions in response to the model
**Primary nature of group task**	Divergent
**Time**	**Preparation**: 5 minutes: Very large representation(s) of a current version of the model/diagram (printed or drawn) taped/affixed to walls or windows<br>**Session**: 30 minutes<br>**Follow up**: Nil
**Materials**	• Very large poster-size representation of model/diagram (printed or drawn)<br>• A3 slips of coloured paper<br>• Several dark felt tip pens (consider having one for each participant)<br>• Blue Tac (if large representations are hung on the wall)
**Inputs**	Causal loop diagram or stock and flow diagram currently being developed in GMB project
**Outputs**	Causal-loop diagram or stock-flow map with stakeholders' action ideas on post-its attached *at the relevant place* on the diagram/model; digital photographs of map/model with post-it comments
**Roles**	• **Facilitator** to introduce the representation to the large group and introduce the guidelines for the activity<br>• **Group facilitator(s)** to manage small group discussions and questions<br>• **Recorders** to document the small and large group discussions
**People needed in the room**	• Participants
**Steps**	1. Ask groups to take some time to identify as many actions as they can that would impact the model from the previous exercise.<br><br>• Participants are given the following instructions, in conjunction with a slide, highlighting the Meadows (1999) leverage points.<br><br>We would now like you to take some time, and use the diagram to help identify as many possible actions to improve this system as you can.<br><br>You can develop interventions that might impact variables directly – for example you might find a way to decrease (example variable). This might be an ineffective way to intervene, however, as it only addresses one symptom of the problem. As we can see there are a number of other variables which are connected to this one, and if they are not addressed as part of the intervention, our success may be limited as we have ignored several of the causes of this variable.<br><br>You might develop ideas that impact on a connection – for example, you might come up with a way to create a new link

	between two variables which were previously disconnected (give example).<br><br>You can also consider interventions which strengthen a connection – for example, a particular action might strengthen the connection between (give example)<br><br>Finally, you can consider interventions which would impact either the rules that the system is governed by, or the goals that the system is trying to achieve – for example, organizational policies could restrict or alter the behaviour of particular variables we have identified in the system, or we may intervene to change the goals that the system as a whole is working towards. These can be the most difficult action ideas to come up with – but they can also be the most effective.<br><br>When considering these action ideas, please write a short, one sentence description of the action idea on your slip of A5 paper. If you can, have a look at the large map in front of you, and see if you can create a small sketch on your A5 page of how your action idea would "fit into" the CLD. Once you have an A5 slip with a describing sentence and an action idea sketch, please place/stick it on the large map in front of you, close to the variables which are most relevant to the action idea.
**Evaluation criteria**	The exercise leads to a rich list of potential actions, which have been identified by the community participants.<br>Participants are energized by the process of offering their potential solutions.<br>The group has developed some understanding of how to place intervention ideas within the causal map.
**Author(s)**	Unknown
**History**	
**Revisions**	
**References**	Meadows, D. 1999. Leverage points: places to intervene in a system. Hartland VT: The sustainability institute.
**Notes**	

Prioritisation of Action Ideas

**Context**	After individual action ideas have been developed.
**Purpose**	To prioritise potential actions in response to the model
**Primary nature of group task**	Convergent
**Time**	**Preparation**: Nil<br>**Session**: 30 minutes<br>**Follow up**: 5 minutes (entering prioritized actions into list)
**Materials**	Butchers paper for themes
**Inputs**	Participants' action ideas from previous activity (Action Ideas script)
**Outputs**	List of 10-20 action ideas, in priority order, as determined by participants
**Roles**	• **Facilitator** to introduce the representation to the large group and introduce the guidelines for the activity<br>• **Group facilitator(s)** to manage small group discussions and questions
**People needed in the room**	• Participants
**Steps**	1. Room facilitator explains to participants that we are now going to move on from generating action ideas to prioritizing and organizing the ideas which have been generated so far.<br><br>2. The room facilitator explains that there will be two rounds of prioritization on the individual tables, before we reconvene as a whole room to share the best 30 ideas as the final output of the workshop.<br><br>3. The room facilitator explains to participants that the prioritization process will revolve around a) the feasibility and b) the impact of each idea. Participants are encouraged to consider feasibility and impact in the context of the evidence reviews which have been presented so far.<br><br>4. The room facilitator explains that the table facilitators will now work with participants on the prioritization process.<br><br>5. The table facilitators open the discussion with participants on their table. Instruction is given to work in groups of three, and that each participant will get the chance to explain their "best" action ideas to the other two in their group.<br><br>6. After participants have had time to share their action ideas in groups of three, instruction is given to consider how impactful and feasible each of their action ideas are, and identify the top 5 most feasible and impactful action ideas from the group of three.

	7. When each group of three has identified its top 5 ideas, the table facilitator brings the three groups back together as a whole table of 9, and explains that the ideas must now be prioritized into a “top 3” for the table.<br><br>8. Each group of three will explain their top two priority action ideas in a round-robin fashion. If an idea has been covered by another group of three, the following group will “skip” the idea, and present the next idea from their top 5.<br><br>9. After sharing their top two ideas per group of 3, the table decides which three ideas represent the most feasible, and impactful, ideas for the table as a group of 9.<br><br>10. After participants have reached the end of the available time, the room facilitator will reconvene the room as a whole, and explain that the final round of prioritization is about to begin.<br><br>11. The room facilitator gives each table the chance to share one action idea at a time, in a round robin fashion, until each table has shared their top 2-3 ideas.<br><br>12. As the table’s ideas are being shared, the A5 action idea slips are being collected from the tables, and stuck to the wall in their relevant theme<br><br>13. Next to each theme, participants are given the chance to “sign up” to working groups on butcher paper attached to the theme.
**Evaluation criteria**	The exercise leads to a prioritised list of potential actions, which have been identified by the community participants.<br>Participants have engaged with each other, and collaboratively discussed action ideas.
**Author(s)**	Unknown
**History**	
**Revisions**	
**References**	Meadows, D. 1999. Leverage points: places to intervene in a system. Hartland VT: The sustainability institute.
**Notes**	

Dots

**Description**	Participants prioritise results from a previous exercise (e.g. graphs over time or action ideas) by placing adhesive dots beside each item.
**Context**	There are many times during GMB sessions where it is important to prioritize or reduce the number of items the group is working on. This might be to choose the top « X » Behavior Over Time Graphs (BOTGs) for inclusion in the model, or to reduce down to a prioritized list of action ideas.
**Purpose**	• To sift through many possible choices and select those most important to the participant group.
**Primary nature of group task**	• Evaluative
**Time**	**Preparation:** 5 minutes to cut up adhesive stickers for each participant<br><br>**Session**: Depending on the size of the group and # of dots given, this can take 5-15 minutes. Consider doing this at the beginning of a break to save time.
**Materials**	• Three to five dots per participant depending on the packaging of adhesive dots
**Inputs from other scripts**	• An array of items to vote on with dots, for example, a set of behavior over time graphs or action ideas
**Outputs from this script**	• Prioritized choices
**Modeling team roles**	• **Facilitator** to introduce the exercise
**People in the room**	• Participants<br>• All members of the core modeling team
**Steps**	1. The **facilitator** gives every participant the same number of dots.<br>2. The **facilitator** instructs **participants** to place their dots beside the items they think are most important to them. They can distribute the dots any way they want (e.g. put all of them on behavior over time graph or spread dots out across several graphs).<br>3. The **facilitator** tallies the dots beside each item to create a ranked list of importance.
**Evaluation criteria**	• Participants have prioritized their choices.<br>• Participants have achieved consensus on the most important items.
**Author(s)**	Unknown
**History**	Unknown
**Revisions**	Documented March 6, 2012 by Timothy Hower and Peter Hovmand

**References**	NA
